# Animal Health Professionals’ Knowledge, Risk Perception and Preventive Practices Towards Zoonotic Infections in Cameroon

**DOI:** 10.1002/puh2.70080

**Published:** 2025-08-01

**Authors:** Victor Ngu Ngwa, Jarvis Dongmo Bouna, Lorucha Amatama Mouyobo, Frédéric Moffo, Mohamed Moctar Mouliom Mouiche, Herman Okah‐Nnane, Justin Kouamo

**Affiliations:** ^1^ Department of Microbiology and Infectious Diseases, School of Veterinary Medicine and Sciences University of Ngaoundéré Ngaoundere Adamawa Cameroon; ^2^ Department of Pharmacy, Pharmacology and Toxicology, School of Veterinary Medicine and Sciences University of Ngaoundéré Ngaoundere Adamawa Cameroon; ^3^ Institute of Agricultural Research For Development Veterinary Research Laboratory Wakwa Regional Center Ngaoundéré Adamawa Cameroon; ^4^ Department of Pathology and Surgery School of Veterinary Medicine and Sciences University of Ngaoundéré Ngaoundere Adamawa Cameroon

**Keywords:** Cameroon, knowledge, para‐veterinarians, preventives practices, risk perception, veterinarians, zoonoses

## Abstract

**Introduction:**

Animal health professionals act as the first line of defence in zoonosis transmission. By handling animals on a daily basis, they are at risk of contracting zoonotic diseases. However, the lack of data on animal health professional risk perception on zoonotic disease and preventive practices may hamper decision making and policies to fight against transmissible disease at the human‐animal and environmental interface.

**Methods:**

A cross‐sectional survey was conducted between July and December 2022 to investigate the knowledge, risk perceptions and preventive practices towards zoonotic infections among 272 selected veterinarians (81) and para‐veterinarians (191) in four regions of Cameroon. Data collection was conducted using questionnaires, and responses were recorded into a binary scale. An ANOVA test was used to assess significant differences in mean knowledge, practice and zoonotic risk perception scores between the two groups of participants, whereas linear regression was done to explore the relationship between demographic characteristic and the knowledge, practice and risk perception.

**Results:**

Overall, a mean knowledge score of 0.79 ± 0.15 towards zoonosis was obtained. Gender and educational level significantly (*p* < 0.05) influence the knowledge scores on zoonosis. More than half (60.5%) of veterinarians perceived wild animal species as the highest zoonotic disease transmitter, whereas 50.26% of para‐veterinarians considered pet animals as the highest risk transmitter species. A low risk of contracting a zoonotic infection during assistance in parturition and when handling apparently healthy animals was considered by all respondents. More veterinarians were reported using adequate personal protective equipment for surgery (17.3%), necropsy (4.7%) and delivery (11.1%) than the para‐veterinarians. A significant association (*p* < 0.05) among regions, service sector, risk perception when handling apparently healthy animals and when getting in contact with animal saliva and animal faeces and preventive practices against zoonotic infection risks was observed. This study reveals that the more participants perceive a risk of contracting a zoonotic infection, the more an adoption towards the right attitude during clinical practice (*r* = 0.20).

**Conclusion:**

Reducing the risk of occupationally acquired zoonotic infections requires continuous education and sensitisation of all stakeholders coupled with appropriate infection prevention and control measures. Thus, the implementation of a One Health (OH) strategy as a sustainable solution for controlling zoonotic infection outbreaks through the enhancement of the collaboration among the key OH stakeholders will improve on the OH system's efficiency.

## Graphical abstract

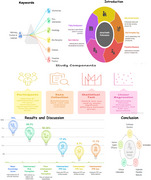

## 1 Introduction

The One Health (OH) concepts that highlight critical roles in addressing world health challenges have been endorsed by major international bodies [1, 2]. The benefits of the OH approaches in areas of detection, prevention and control of infectious diseases at the human–animal–environment interface have been discussed [3]. Nowadays, the OH concept is increasingly recognised as essential for strengthening health systems worldwide, particularly against zoonotic and other emerging disease threats [4]. The pandemic of COVID‐19 and recent outbreaks of avian influenza, Ebola virus, Zika virus and other emerging and re‐emerging zoonotic pathogens have served as a reminder that human beings are always exposed to infectious threats [5]. It is known that about 60% of pathogenic infectious organisms to humans and 75% of all emerging infectious diseases to humans are zoonotic [6, 7, 8]. Due to the potential danger and widespread exposure that can occur with such pathogens, they represent a global public health security at the animal, human and environmental interface [9, 10].

Cameroon shares many drivers of zoonotic diseases with other countries in the West and Central Africa region, considered one of the world's hotspots for emerging and re‐emerging diseases [11]. Key factors contributing to the risk of zoonotic disease transmission in Cameroon include the geographical location, increasing agricultural intensification and rich biodiversity, which foster close human–animal–environment interactions [12]. Furthermore, certain cultural practices, such as keeping animals close to homes and allowing them to roam freely, which encourage close human–animal contact; consuming undercooked bushmeat; drinking raw blood from animals; and handling dead animals found in the forest zone [13] in association with limited veterinary and public health facilities, may aggravate the risk.

The priority zoonoses in Cameroon include bovine tuberculosis (found mostly in the bimodal rainforest and high savannah zones), high pathogenic avian influenza (monomodal rainforest and highlands zones) and rabies (Sudano‐Sahelian zone) [14]]. Other zoonoses of importance with sporadic outbreaks in Cameroon include anthrax, brucellosis, Ebola, Lassa fever, monkeypox and trypanosomiasis.

Over the years, Cameroon has reported several outbreaks of highly pathogenic avian influenza, significantly impacting poultry production and livelihoods of farmers [15, 16]. Anthrax is sporadically reported, resulting in deaths in animals mostly [17]. Bovine and human tuberculosis are constant threats, especially in the bimodal rainforest and high savannah zones [18], and rabies remains a significant public health threat, especially in the Savanna Sahelian zone [19].

The permanent contact with animals during their daily activities increases the risk for animal health professionals, who represent one of the most vulnerable populations to zoonotic pathogen infection [20, 21]. They and their assisting workers are usually the first to encounter potentially infected animals during clinical investigation, and they are at risk of developing zoonotic infections [22].

In Cameroon, the use of protective clothing and sanitisers is employed as a preventive measure by animal health professionals to mitigate risks of infections, but their effectiveness depends on the risk perceptions and appropriate applications during veterinary procedures. Over the years, it has been observed that animal health professionals are at a high risk of many zoonotic infections at work through the possible nature of exposures, such as contamination with secretions and bites and scratches of animals. A high percentage (60%–65%) of veterinarians has been reported to have contracted zoonotic diseases through similar means of exposure in the United Kingdom [23] and in South Africa [24].

Some studies in Cameroon [25, 26] revealed that high‐risk behavioural practices and low‐risk perception are among the main factors facilitating the transmission of zoonosis. In most of the Low‐ and Middle‐Income countries, including Cameroon, the risk perception of animal health professionals about zoonotic disease and the implementation of preventive measures in their daily practice is still to have the appropriate desired attention. Furthermore, a good understanding of the protective behaviours against infectious diseases by animal health professionals is important to establish effective first‐line public health and preventive practices programs on zoonoses. Therefore, this study aimed to understand the zoonotic risk perception and infectious disease protective practices among animal health professionals to identify gaps and opportunities for enhancing OH strategies for zoonoses control and prevention programs in Cameroon. In addition to providing valuable insights for low‐ and middle‐income countries, the results are expected to contribute to a broader understanding of the notion of OH and zoonotic disease management.

## 2 Materials and Methods

### 2.1 Study Area

**FIGURE 1:**
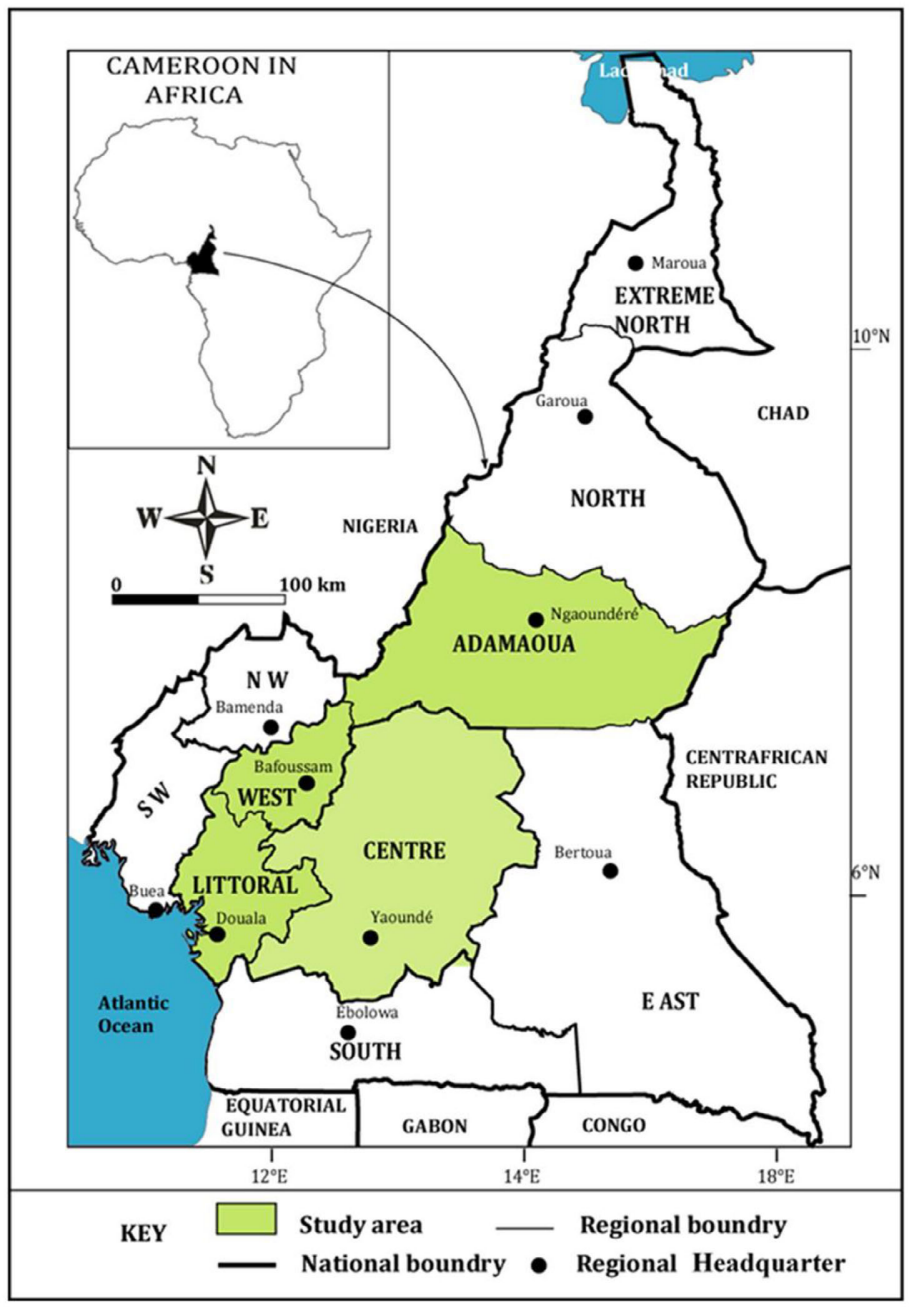
Map showing the study regions (Adamawa, West, Littoral and Centre) in Cameroon.

The study was carried out from July to December 2022 in Adamawa (6°49′59″ LN and 13°15′0″ LE), Centre (3°52′–6°14′ LN and 11°31′–12°93′ LE), Littoral (4°03′–4°90′ LN and 9°42′–10°43′ LE) and West (5°02′–5°39′ LN and 9°40′–11°11′ LE) regions of Cameroon (Figure 1). These four regions host about 75% of stakeholders in the veterinary medicine sector in Cameroon (NVAC 2020) [27]

### 2.2 Study Design and Sampling Procedure

The methodological approach consisted of a cross‐sectional questionnaire‐based survey. The target population was animal health professionals practicing in the study areas within the study period. A list of private and public veterinary clinics, pharmacies, laboratories and abattoirs located in the study areas was obtained from the Department of Veterinary Services of the Ministry of Livestock, Fisheries and Animal Industries in Cameroon. All these veterinary structures were approached, and veterinarians and their assistants were asked to take part in the survey. Veterinarians whose daily activities did not involve contact with animals or animal tissues were not included in the survey. Only veterinarians and para‐veterinarians who willing to participate in the survey were included in the study.

Previous studies on zoonotic risk perception and infectious disease protective behaviours in animal health professionals guided the development of the survey questionnaire [28, 29, 30]. The questionnaire was developed in English and French and contained mostly close‐ended questions to ease data processing, minimise variation and improve precision of responses (Thrusfield 2009) [31]. Besides the demographic characteristics of respondents (age, sex, type of animal practice, years of experience and level of education), three sub‐themes were investigated, namely, knowledge of zoonosis, risk perception of zoonotic diseases and preventive practices. The responses included dichotomous and categorical outcomes (yes/no), ordinal outcomes (3‐point Likert scale type: low/moderate/high) and open‐ended questions.

The questionnaire was pre‐tested with 10 animal health professionals before the study and revised accordingly. The implementation was a face‐to‐face interview with the respondents after obtaining their verbal informed consent.

### 2.3 Data Management and Statistical Analysis

Data were entered in a Microsoft Excel spreadsheet (Microsoft Corporation, Redmond, WA, USA) and critically reviewed by two supervisors before being exported and analysed using R software (version 3.6.1). Descriptive statistics were used to express results as frequencies and proportions and compared between veterinarians and para‐veterinarians with the chi‐square test or the Fisher exact test (when the expected frequencies were lower than 5). Data on knowledge of zoonosis were recoded into binary outcomes, with 1 representing sufficient knowledge of zoonosis, whereas 0 represented insufficient knowledge. The sum of responses recorded for each participant was then divided by the total number of items within the knowledge category to arrive at a percentage of correct answers (Caudell et al. 2020) [32]. A one‐way ANOVA was performed to identify factors associated with sufficient knowledge on zoonosis. Reported use of PPE (personal protective equipment) for each scenario presented to the respondents was compared to the recommendation of the National Association of State Public Health Veterinarians guidelines in different clinical scenarios [29] and designated as ‘adequate’ or ‘inadequate’ if they comply or not with the minimum PPE recommended. To identify factors associated with appropriate use of PPE, each respondent was assigned a score that reflected the stringency of his protection for all scenarios presented. For each scenario, a score of 1 was assigned when respondents use adequate PPE, and a score of 0 when the PPE is inadequate. The sum of responses recorded for each participant was then divided by the total number of scenarios to arrive at a percentage of correct answers. To keep them as binary variables, the score range was categorised into ‘Poor’ (less than 50%) and ‘Satisfactory’ (50% or more). Associations between the outcome and explanatory variables were first subjected to univariable analyses using chi‐square tests (Dohoo et al. 2009) [33]. All factors found to be statistically significant were subsequently analysed using binary logistic regression. A *p* value < 0.05 was considered statistically significant.

## 3 Results

### 3.1 Demographic Characteristics of Animal Health Professionals

Out of the 272 respondents interviewed, more than half were male (52.9%), aged between 18 and 34 years old (50.4%). They were mostly located in Littoral (35.3%) and Centre (33.5%) regions. More than half of the respondents (54.0%) were general practitioners with less than 5 years of professional experience (46.0%); overall, 44.5% were working in the public sector, whereas 43.4% were working in the private sector. In addition, out of the 128 female respondents, more than half were young, aged between 18 and 34 years old (83/128 [64.8%]) with less experience (70/125 [56%]) in handling pets and farm animals (Table 1).

**TABLE 1:** Demographic characteristics of animal health professionals surveyed in the Adamawa, Centre, Littoral and West regions of Cameroon.

Factors	Variables	Para veterinarians (%)	Veterinarians (%)	Total (%)
Age	18–34	80 (42.1)	57 (70.4)	137 (50.4)
	35–54	106 (55.8)	22 (27.2)	128 (47.1)
	55±	5 (2.1)	2 (2.5)	7 (2.6)
Gender	Female	88 (46.1)	40 (49.4)	128 (47.1)
	Male	103 (53.9)	41 (50.6)	144 (52.9)
Region	Adamawa	27 (14.1)	14 (17.3)	41 (15.1)
	Centre	61 (31.9)	30 (37.0)	91 (33.5)
	Littoral	73 (38.2)	23 (28.4)	96 (35.3)
	West	30 (15.7)	14 (17.3)	44 (16.2)
Experience (in years)	[0–5]	68 (35.6)	57 (70.4)	125 (46.0)
	[6–10]	69 (36.1)	9 (11.1)	78 (28.7)
	[11–15]	19 (9.9)	3(3.7)	22 (8.1)
	[16–20]	25 (13.1)	3 (3.7)	28 (10.3)
	[21±]	10 (5.2)	9 (11.1)	19 (7.0)
Specialisation	Pets	30 (15.8)	13 (16.0)	43 (15.8)
	Pets and farm animals	102 (53.7)	45 (55.6)	147 (54.0)
	Farm animals	58 (30.5)	23 (28.4)	82 (30.2)
Service sector	Private clinic	46 (60.2)	73 (90.1)	119 (43.7)
	Public clinic	115 (24.1)	6 (7.4)	121 (44.5)
	Abattoir	26 (13.6)	0 (0.0)	26 (9.6)
	Laboratory	4 (2.1)	2 (2.5)	(2.2)
Professional status	Consultant	0 (0.0)	1 (1.3)	1 (0.4)
	Employee	185 (96.9)	48 (59.3)	(85.7)
	Manager	4 (2.1)	26 (32.1)	30 (11.0)
	Intern	2 (1.0)	6 (7.4)	8 (2.9)

### 3.2 Knowledge of Zoonosis by Animal Health Professionals

Almost 99% of respondents had heard about zoonosis. Veterinarians (93.8%) were more acknowledged that zoonotic pathogens can also be transmitted from human to animal than para‐veterinarians (78.5%). On the list of 13 infectious pathogens provided with 11 zoonotic pathogens, 62.5% of respondents were able to recognise at least half of the zoonotic pathogens of the list (at least 6 zoonoses). However, the proportion of veterinarians (82.7%) that could rightly identify at least six zoonoses in the list was significantly (*p* < 0.05) higher than that of para‐veterinarians (53.9%) (Table 2).

**TABLE 2:** Knowledge levels of animal health professionals on zoonoses in Cameroon.

Variables	Professional grade	Yes (*N*/%)	No (*N*/%)	*p* value
Have heard about zoonosis	Veterinarians	80 (98.8)	01 (1.2)	0.89
	Para‐veterinarians	189 (99)	02 (1)	
Correctly define zoonosis	Veterinarians	80 (98.8)	01 (1.2)	0.89
	Para‐veterinarians	189 (99)	02 (1)	
Zoonosis can be transmitted by apparently healthy animals	Veterinarians	76 (93.8)	05 (6.2)	0.21
	Para‐veterinarians	170 (89.0)	21 (11.0)	
Zoonosis can be transmitted from human to animal	Veterinarians	76 (93.8)	05 (6.2)	0.002
	Para‐veterinarians	150 (78.5)	41 (21.5)	
Zoonosis can be transmitted from human to human	Veterinarians	62 (76.5)	19 (23.5)	0.25
	Para‐veterinarians	133 (69.6)	58 (30.4)	
Recognised at least 6 zoonoses over 11	Veterinarians	67 (82.7)	14 (17.3)	<0.0005
	Para‐veterinarians	103 (53.9)	88 (46.1)	

The mean knowledge scores of respondents towards zoonosis of 0.79 ± 0.15 were obtained. Gender and educational level of the respondents significantly (*p* < 0.05) influence the knowledge scores on zoonosis. The mean score observed with males (0.82 ± 0.14) was significantly higher (*p* < 0.05) than in females (0.76 ± 0.16). The mean score of veterinarians (0.83 ± 0.12) towards knowledge of zoonosis was significantly (*p* < 0.05) higher than that observed with para‐veterinarians (0.78 ± 0.16) (Table 3).

**TABLE 3:** Mean knowledge scores according to socio‐demographic characteristics of animal health professionals in Cameroon.

Variables	Mean knowledge scores	*p* value
Age		
18–34	0.77 ± 0.16	0.14
35–54	0.81 ± 0.14	
55+	0.83 ± 0.12	
Gender		
Female	0.76 ± 0.16	0.02
Male	0.82 ± 0.14	
Experience (in years)		
[0–5]	0.79 ± 0.15	0.30
[6–10]	0.77 ± 0.16	
[11–15]	0.82 ± 0.14	
[16–20]	0.80 ± 0.17	
[21±]	0.85 ± 0.12	
Specialisation		
Pets	0.83 ± 0.17	0.20
Pets and farm animals	0.78 ± 0.17	
Farm animals	0.79 ± 0.13	
Region		
Adamawa	0.76 ± 0.17	0.45
Centre	0.81 ± 0.16	
Littoral	0.79 ± 0.14	
West	0.79 ± 0.15	
Grade		
Veterinarians	0.83 ± 0.12	0.009
Para‐veterinarians	0.78 ± 0.16	
Overall	0.79 ± 0.15	

### 3.3 Risk Perception About Zoonosis

**FIGURE 2:**
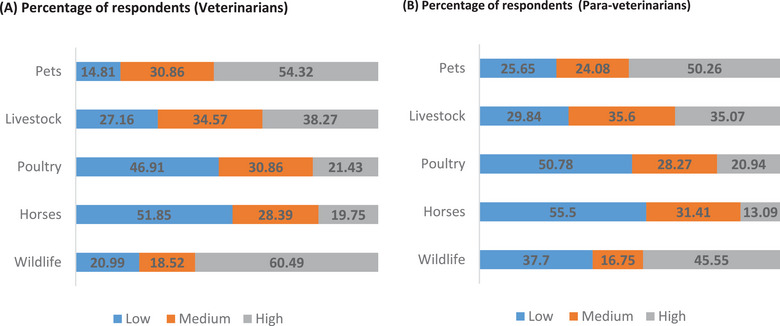
Probability of being infected when handling different animal species as perceived by veterinarians and para‐veterinarians in Cameroon.

**FIGURE 3:**
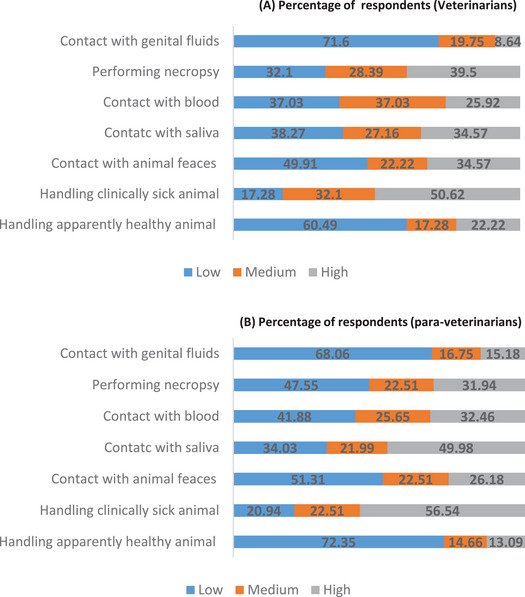
Probability of being infected when managing some clinical cases as perceived by veterinarians and para‐veterinarians in Cameroon.

As for the animal health professionals’ perceptions on the risk of exposure to zoonotic pathogens when handling different animal species, more than half (60.5%) of veterinarians perceived the risk to be high with wild animals, whereas more than half (50.26%) of para‐veterinarians perceived the risk to be high with pets. Horses were perceived as animals with the lowest risk for zoonotic disease transmission by more than half of the two subgroups of respondents (Figure 2). Regarding the risk of contracting a zoonotic disease when performing veterinary procedures and getting into contact with animal fluids, half of veterinarians (50.62%) and para‐veterinarians (56.54%) perceived handling clinically sick animals as a high‐risk situation regarding zoonotic infection. The aspect of the job considered the lowest risk of exposure to zoonoses was during parturition for 71.6% of veterinarians and handling apparently healthy animals for 72.35% of para‐veterinarians (Figure 3).

### 3.4 Infection Mitigation Practices (IMP)

As concerns the zoonotic IMPs, veterinarians (55.6%) were significantly (*p* < 0.05) more aware of how to counteract the common professional zoonosis by vaccination than para‐veterinarians (31.9%). Moreover, veterinarians (44.4%) were significantly observed to separate the working area from eating area more than para‐veterinarians (29.3%). Regarding hand hygiene, use and disposal of syringes, isolation of sick animals and use of PPE, variable proportions of respondents indicated that they applied significant preventive practices with no significant difference between veterinarians and para‐veterinarians (Table 4).

**TABLE 4:** Animal health professionals’ preventive practices to mitigate zoonotic diseases risk in Cameroon.

Practices	Professional grade	Yes (*N*/%)	No (*N*/%)	*p* value
Vaccination against some zoonotic diseases	Veterinarians	45 (55.6)	36 (44.4)	0.0002
	Para‐veterinarians	61 (31.9)	130 (68.1)	
Always washing hand before handling animals	Veterinarians	68 (84.0)	13 (16.0)	0.37
	Para‐veterinarians	169 (88.0)	23 (12.0)	
Separate the eating area from the working area	Veterinarians	36 (44.4)	45 (56.6)	0.01
	Para‐veterinarians	56 (29.3)	135 (70.7)	
Always recap needles before disposal	Veterinarians	47 (58.0)	34 (42.0)	0.79
	Para‐veterinarians	114 (59.7)	77 (40.3)	
Proper elimination of needles	Veterinarians	44 (54.3)	37 (45.7)	0.89
	Para‐veterinarians	102 (53.4)	89 (46.6)	
Sterilisation and reutilisation of needles	Veterinarians	11 (13.6)	70 (86.4)	0.39
	Para‐veterinarians	34 (17.8)	157 (82.2)	
Isolation of sick animals	Veterinarians	17 (21.0)	64 (79.0)	0.99
	Para‐veterinarians	40 (20.9)	151 (79.1)	
Use personal protective equipment	Veterinarians	48 (59.3)	33 (40.7)	0.13
	Para‐veterinarians	94 (49.2)	97 (50.8)	

**TABLE 5:** Use of minimal personal protective equipment (PPE) as a preventive measure by animal health professionals in Cameroon with respect to clinical scenarios.

Procedure	Professional grade	Adequate	Inadequate	*p* value
Handling apparently healthy animal	Veterinarians	39 (48.1)	42 (58.9)	0.18
	Para‐veterinarians	109 (57.1)	82 (42.9)	
Performing surgery	Veterinarians	14 (17.3)	67 (82.7)	0.01
	Para‐veterinarians	0 (0.0)	191 (100.0)	
Performing necropsy	Veterinarians	9 (11.1)	72 (88.9)	0.06
	Para‐veterinarians	0 (0.0)	191 (100.0)	
Assisting in animal parturition	Veterinarians	2 (2.5)	79 (97.5)	0.99
	Para‐veterinarians	0 (0.0)	191 (100.0)	
Handling faeces/urine samples	Veterinarians	37 (45.7)	44 (54.3)	0.37
	Para‐veterinarians	76 (39.8)	115 (60.2)	
Treating animals with skin diseases	Veterinarians	44 (54.3)	37 (45.7)	0.21
	Para‐veterinarians	88 (46.1)	103 (53.9)	
Treating animals with respiratory diseases	Veterinarians	41 (50.6)	40 (49.4)	0.18
	Para‐veterinarians	80 (41.9)	111 (58.1)	
Treating animals with gastrointestinal diseases	Veterinarians	32 (39.5)	49 (60.5)	0.10
	Para‐veterinarians	56 (29.3)	135 (70.7)	

### 3.5 Reported Use of PPE

As concerning the usage of PPE, less than one‐third of the veterinarians reported using adequate PPE for surgery (17.3%), necropsy (4.7%) and delivery (11.1%) (Table 5). Main reasons are due to the poor risk perception factor and unavailability of this equipment in their structures as a result of management challenges. In handling healthy animals, treating skin and respiratory diseases, at least 50% of the veterinarians declare using adequate PPE. Regarding the factors influencing appropriate use of PPE, univariate analysis with the chi‐squared test identified regions and the service sector as high potential risk points when handling apparently healthy animals and when getting in contact with animal saliva and animal faeces as independent factors significantly associated with preventive practices against zoonotic infection risks (*p* < 0.05). The univariate logistic regression indicated that animal health professionals working in laboratories were 10 times more stringent in PPE use than those working in clinics. Animal health professionals who perceive the risk of handling apparently sick animals and contact with animals’ faeces as high were, respectively, 3.74 and 2.37 times more stringent in PPE use than those who perceive the risk as low (Table 6).

**TABLE 6:** Factors associated with stringency in the use of personal protective equipment (PPE) by animal health professionals in Cameroon.

Factors	Poor PPE use	Satisfactory PPE use	Odds ratios	95% CI	*p* value
**Region**					
Littoral	59 (31.9)	37 (42.5)	1	/	/
Centre	60 (32.4)	31 (35.6)	0.82	0.45–1.50	0.52
Adamawa	27 (14.6)	14 (16.1)	0.83	0.38–1.78	0.62
West	39 (21.1)	05 (5.7)	0.20	0.07–0.56	0.02
**Service**					
Private clinic	81 (43.8)	38 (43.7)	1	/	/
Abattoir	16 (8.6)	10 (11.5)	1.33	0.55–3.20	0.52
Public clinic	81 (43.8)	38 (43.7)	0.83	0.48–1.48	0.51
Laboratory	01 (0.5)	5 (5.7)	10.66	1.20–9440	0.03
**Risk perception when handling apparently sick animals**					
Low	143 (77.3)	44 (50.6)	1	/	/
Moderate	22 (11.9)	20 (23.0)	2.95	1.48–5.91	0.002
High	20 (10.8)	23 (26.4)	3.74	1.88–7.44	0.0002
**Risk perception when getting into contact with animal saliva**					
Low	69 (37.3)	27 (31.0)	1	/	/
Moderate	49 (26.5)	15 (17.2)	0.78	0.38–1.62	0.51
High	67 (36.2)	45 (51.7)	1.72	0.96–3.07	0.07
**Risk perception when getting into contact with animal faeces**					
Low	99 (53.5)	34 (39.1)	1	/	/
Moderate	43 (23.2)	18 (20.7)	1.22	0.62–2.39	0.56
High	43 (23.2)	35 (40.2)	2.37	1.31–4.28	0.04

## 4 Discussion

Animal health professionals are always at risk of being exposed to or contracting infectious diseases. However, assessing factors which might increase the risk among animal health professionals is crucial. Therefore, this study was carried out with the aim of assessing the knowledge, perceptions about risks and different control measures with respect to zoonotic infections in Cameroon.

Results of the investigation of animal health professionals regarding zoonosis in Cameroon indicated that almost all of the respondents could rightly identify or explain zoonoses. This observation differs from that reported by Alhaji et al. [30] who acknowledged that a more significant number of Nigerian veterinarians were capable of correctly defining zoonoses than para‐veterinarians; that of Zhang et al. [34], whose results revealed a low awareness and poor knowledge of zoonoses by veterinary field staff in Tanzania; and also the study of Majiwa et al. [35] in Kenya, where participants demonstrated insufficient knowledge of zoonosis. The good knowledge of zoonosis obtained in this study is certainly associated with the level of education of both groups of respondents, as confirmed by the significant association between the knowledge score and the level of education. This observation collaborates with the findings of Ȯzlü et al. [36] in Turkey that revealed that the awareness of zoonotic diseases was positively associated with higher educational levels. Furthermore, this study also revealed that a non‐negligible proportion of the respondents could not recognise some zoonotic infections among a group of diseases. This corroborates with the findings of Majiwa et al. [35] in Kenya and Alhaji et al. [30] in Nigeria, where a significant proportion of respondents had difficulty in classifying some pathogens as zoonoses.

The risk perception and knowledge on zoonotic infections are directly proportional to some protective measures adopted by animal health professionals. In this study, a higher proportion (86.6%) of participants were declared practicing hand hygiene as a protective measure compared to the 75% recorded in a previous study in Arizona, USA [37], and this could be due to the fact that, though not taken into consideration during this survey, animal health professionals make lesser use of gloves. Our findings in this study also revealed that the majority (55.5%) of veterinarians have received at least one type of vaccine before they began their activities, as opposed to 31.3% for para‐veterinarians. The relatively high proportion of vaccinated veterinarians could probably be as a result of the consideration of zoonoses as well as their consequences, especially when they just left school. The disconnection among the knowledge, risk perception on zoonoses and the high proportion of undated vaccines observed within the animal health professionals may reflect that veterinarians are just hoping for the best when it comes to contracting zoonoses. In either case, this attitude calls for a sensitisation on the importance of zoonoses control, especially for endemic zoonotic diseases, in order to improve immunologic practice. Veterinarians have a moral and legal responsibility to provide a safe workplace for their staff [38]. In the present survey, 66.2% of respondents reported not having a separate room for eating. This perceived lack of control measure in relation to a risk is known to reduce the uptake of protective behaviours [39, 40].

The present study revealed that a high proportion of para‐veterinarians (67.5%) and veterinarians (64.4%) frequently engaged in recapping needles, in accordance with results obtained in a similar study carried out in the USA [41]. This finding is different from the study of Alhaji et al. [30] in Nigeria, where a reasonably higher proportion of veterinarians than para‐veterinarians were found to be recapping needles prior to disposal. Such activities increase the risk of percutaneous injury, which is a favourable route for zoonotic disease transmission. The Compendium of Veterinary Standard Precautions for Zoonotic Disease Prevention in Veterinary Personnel states that ‘needles should not be recapped’ [42]. This practice implemented by a large number of veterinarians and para‐veterinarians was considered unacceptable in a previous study [43], as one accidental self‐injection directly resulted in a spontaneous abortion. Reports from a previous study [36] revealed that 19 of 199 (10%) veterinarians in Wisconsin reported needle stick exposures during administration of *Mycobacterium avium* subsp. *paratuberculosis* bacterin [44]. It should be noted that, occasionally, the use of live vaccines may prove infectious for the operators [44, 45]. Poor information about zoonoses and the different risk factors on contracting infectious diseases, as well as ignorance on veterinary standard precautions during work, enhances negative conduct, and the consequences of this ignorance could be a resulting increase in animal health professionals’ contracting an infection [46, 47].

Even though previous reports classify PPE as the least effective pathogen infectious control method [48], the nature of the training veterinarians receive and the daily high risk in their work procedures expect them to use complete PPE kits [28]. Furthermore, adequate protective practices have been reported to reduce human exposure to zoonotic pathogens [49]. However, veterinarians’ awareness on the existence and consequences of zoonoses that should guarantee the use of adequate PPE was poorly observed in this present study. This indicates that personal choices for PPE vary greatly among the clinical cases at hand, as reported by Robin et al. [29]. Furthermore, contrary to the results obtained by Venkat et al. [37], in which 96% of respondents used PPE during surgery and 94% during necropsy, data analysis found in this study revealed a very low percentage of adequate usage of PPE during surgery (17.3%), necropsy (4.7%) and parturition (11.1%), giving the impression that there is little or no risk of contracting zoonotic infection during these procedures. Alhaji et al. [30] also observed the use of minimal PPE during high‐risk veterinary procedures in a study in Nigeria. Furthermore, Pappas et al. (2005) [50] reported that minimal PPE is not adequate for protection against emerging and re‐emerging zoonoses because small droplets of body fluids could be released during the handling process. The probable reason for a very low usage of adequate PPE is that some animal health professionals consider zoonotic diseases to be an abstract phenomenon [29]. An appropriate PPE should be selected on the basis of the anticipated level of exposure [51], for some animals infected with gastrointestinal pathogens may remain asymptomatic but shed pathogens in their faeces, urine and saliva [52]. These pathogens could be transmitted through these secretions to other susceptible animals and veterinarians and manifest with symptoms [53]. Awareness and education have been reported as significant factors that influence the use of PPE [28]. Thus, it is important to implement a comprehensive training program for the appropriate use of PPE by animal health professionals in Cameroon, especially in regard to emerging and re‐emerging zoonotic infections.

Results from the association of risk perception and participants’ attitudes in this study reveal that the more participants perceive a risk of contracting a zoonotic infection, the more they will adopt the right attitude during the clinical practice (*r* = 0.20).

The findings of this study have certain limitations. The nature of the data collection strategy is based on face‐to‐face interviews with a high response rate. Though the questionnaire seemed hard to fill, the reasonable number of the items was helpful in obtaining more responses regarding veterinarians and para‐veterinarians behaviour towards knowledge, risk perception and protective measures. These assessments could potentially be affected by social bias. However, qualitative data obtained through responses from interviews formed the basis for dependent variable scores that were well complemented in this study. These scores were useful in evaluating the knowledge and risk perceptions on zoonotic infections and protective practices against exposure to zoonotic pathogens and the perceived benefit of engaging in such practices.

## 5 Conclusion

This study assessed knowledge, risk perception and preventive practices of animal health professionals towards zoonosis in Cameroon. Veterinarians were more acknowledgeable and aware towards the risk of zoonotic transmission than para‐veterinarians. Low proportion of animal health professionals were reported using adequate PPE. Reducing the risk of occupationally acquired zoonotic infections requires continuous education and sensitisation of all animal health professionals coupled with appropriate infection preventive and control measures. Thus, we recommend the implementation of an OH approach strategy as a sustainable solution for controlling zoonotic infection outbreaks by enhancing and strengthening collaboration among the key OH stakeholders in Cameroon so as to improve on the OH system's efficiency.

## Author Contributions

**Victor Ngu Ngwa**: conceptualisation, data curation, formal analysis, funding acquisition, investigation, methodology, project administration, software, supervision, writing – original draft, writing – review and editing. **Jarvis Dongmo Bouna**: conceptualisation, formal analysis, methodology, writing – original draft, writing – review and editing. **Lorucha Amatama Mouyobo**: data curation, formal analysis, investigation, methodology, writing – original draft, writing – review and editing. **Frédéric Moffo**: data curation, formal analysis, writing – review and editing. **Mohamed Moctar Mouliom Mouiche**: data curation, writing – review and editing. **Herman Okah‐Nnane**: formal analysis, writing – review and editing. **Justin Kouamo**: conceptualisation, formal analysis, methodology, supervision, validation, writing – original draft, writing – review and editing.

## Ethics Statement

The studies were conducted in accordance with the local legislation and institutional requirements.

## Consent

The participants provided their written/oral informed consent to participate in this study.

## Conflicts of Interest

The authors declare no conflicts of interest.

## Data Availability

The original contributions presented in the study are included in the article; further inquiries can be directed to the corresponding authors.
